# Impact of scaffold granule size use in Masquelet technique on periosteal reaction: a study in rat femur critical size bone defect model

**DOI:** 10.1007/s00068-020-01516-9

**Published:** 2020-10-06

**Authors:** Maximilian Leiblein, Andreas Winkenbach, Elias Koch, Alexander Schaible, Hubert Büchner, Ingo Marzi, Dirk Henrich, Christoph Nau

**Affiliations:** 1 Klinik für Unfall-, Hand- und Wiederherstellungschirurgie, Klinikum der Johann Wolfgang Goethe-Universität, Frankfurt am Main, Germany; 2 Heraeus Medical GmbH, 61273 Wehrheim, Germany

## Abstract

**Purpose:**

The Masquelet technique for the treatment of large bone defects is a two-stage procedure based on an induced membrane. Compared to mature periosteum, the induced membrane differs significantly. However, both play a crucial role in bone regeneration. As part of a histological and radiological post-evaluation of an earlier project, we analyzed the influence of the granule size of the bone void filler Herafill^®^ on development of periosteum regrowth in a critical size defect.

**Methods:**

We compared three different sizes of Herafill^®^ granules (Heraeus Medical GmbH, Wehrheim) in vivo in a rat femoral critical size defect (10 mm) treated with the induced membrane technique. After 8 weeks healing time, femurs were harvested and taken for histological and radiological analysis.

**Results:**

A significantly increased regrowth of periosteum into the defect was found when small granules were used. Large granules showed significantly increased occurrence of bone capping. Small granules lead to significant increase in callus formation in the vicinity to the membrane.

**Conclusion:**

The size of Herafill^®^ granules has significant impact on the development of periosteal-like structures around the defect using Masquelet’s induced membrane technique. Small granules show significantly increased regrowth of periosteum and improved bone formation adjacent to the induced membrane.

## Introduction

The treatment of large bone defects caused by tumor resections, osteomyelitis, or trauma is still a major issue for surgeons and patients [1]. While autologous bone grafting is still considered as gold standard for the treatment of bone defects, Masquelet et al. described an alternative two-stage procedure based on an induced membrane [2].

In a first step, a fibrous membrane is induced around the defect by implanting a PMMA spacer. Then, in a second step, after 6–8 weeks, the induced membrane is opened, the spacer removed and replaced with either cancellous bone or a scaffold material.

In previous work our group demonstrated that multiple factors influence the development and quality of the induced membrane. We were able to show that different sorts of cement and additive antibiotics have a significant influence on the development of the membrane [3].

While neovascularization and osteogenic activity peaks at 2–4 weeks, it is hypothesized that after 6 weeks, the induced membrane acts as a bioreactor concentrating growth factors and regenerative cells [4].

However, compared to mature periosteum, the induced membrane differs significantly in regard to general structure, degree of vascularization and cellular content [4].

The periosteum is a thin tissue connected to the outer surface of the bone by Sharpey’s fibers [5, 6]. It contains mesenchymal progenitor cells, differentiated osteogenic progenitor cells, osteoblasts, and fibroblasts. With its highly vascularized outer layer, it contributes essentially to a bone’s blood supply [7]. The inner layer is rich of progenitor cells, constantly building and repairing bone [6]. Due to its qualities, the periosteum plays a crucial role in bone regeneration and has, therefore, been used in different approaches for reconstruction of large bone defects. Techniques using a vascularized periosteal flap, creating a periosteal sleeve by segment transport or just using the periosteum as a reservoir of osteogenic cells have been described [8–12].

Despite the significant differences between the induced membrane and periosteum, both promote bone regenerative cells, growth factors and vascularization [4, 13].

Herafill^®^ (Heraeus Medical GmbH, Werheim) is a bioresorbable bone void filling material, commercially available in three different granule sizes (0.5–1.0 mm/1.0–3.0 mm/3.0–5.0 mm). The primary component of Herafill^®^ is calcium sulfate, combined with calcium carbonate, triglyceride, and gentamicin [14].

Herafill^®^ is mainly used as a carrier for antibiotics [15], indicated in the context of treatment of osteomyelitis [16, 17], perioperative infections [18] as well as periprosthetic joint infections [19]. Also, excellent properties inducing new bone formation were observed in a clinical trial [20].

In an earlier project, we found that the granule size of Herafill^®^ has significant impact on bone healing in a critical size bone defect in combination with Masquelet technique [14]. In a second study, as part of a histological and radiological post-evaluation based on this already published study, we analyzed the influence of the granule size of Herafill^®^ on the development of periosteum-like structures in the defect.

## Materials and methods

### Ethics and animal care

All animal experiments were performed in accordance with regulations set forth by our institution’s animal care and oversight committee (Project No. FK/K1053 Regierungspräsidium, Darmstadt, Germany) in accordance with German law. Twelve-week-old male Sprague–Dawley rats (Janvier, France), weighing approximately 350–400 g were housed in individual cages, in temperature 21.8 °C), air flow and light (12 h day and 12 h night) controlled rooms and received rat food and water ad libitum. Animals were monitored daily in the postoperative period for signs of pain, discomfort, and complications.

### Biomaterial characterization (Herafill^®^)

Herafill^®^ (Heraeus Medical GmbH, Wehrheim) is a bioresorbable bone void filling material. The primary component of Herafill^®^ is calcium sulfate, combined with calcium carbonate, triglyceride, and gentamicin (Heraeus Medical GmbH, 2017). Three different ranges of granule sizes of Herafill^®^ were used as scaffold (0.5–1 mm, 1–3 mm, 3–5 mm). The material was kindly provided by Heraeus Medical (Wehrheim, Germany).

### Surgical procedure

Surgical procedure was performed as described in earlier work by our group [3, 21–24]. Under general intraperitoneal anesthesia (Ketavet 70 mg/kg and Rompun 10 mg/kg) the right leg was shaved, cleaned and disinfected, and animals were placed in a lateral position. A longitudinal incision was made in the skin and the fascia over the right femur. The biceps femoris and vastus lateralis muscles were separated bluntly exposing the antero-lateral aspect of the femoral bone. A 6-hole, 1.5-mm stainless-steel mini-plate (Synthes, Dubendorf, Switzerland, Compact Hand) was applied to the anterior aspect of the femur shaft and secured in place with two proximal and two distal 1.5 mm cortical screws (Synthes, CompactHand). After stabilization, a critical size defect (CSD), measuring 10 mm, was created in the femur bone shaft, underneath the plate using a Gigli saw (RI-Systems, Davos, Switzerland). The cement was hand mixed according to the manufacturer’s protocol. The bone defect was then filled with PMMA (Palacos) cement and molded into a cylindrical shape. The wound was irrigated with sterile saline, the fascia was re-approximated with interrupted 5–0 Vicryl sutures, and the superficial fascia and skin closed with Prolene 5–0 suture (Ethicon, Germany). Animals were returned to their cages, monitored daily for the occurrence of abnormal behavior or complications and analgesia was given for seven days postoperatively.

**Fig. 1:**
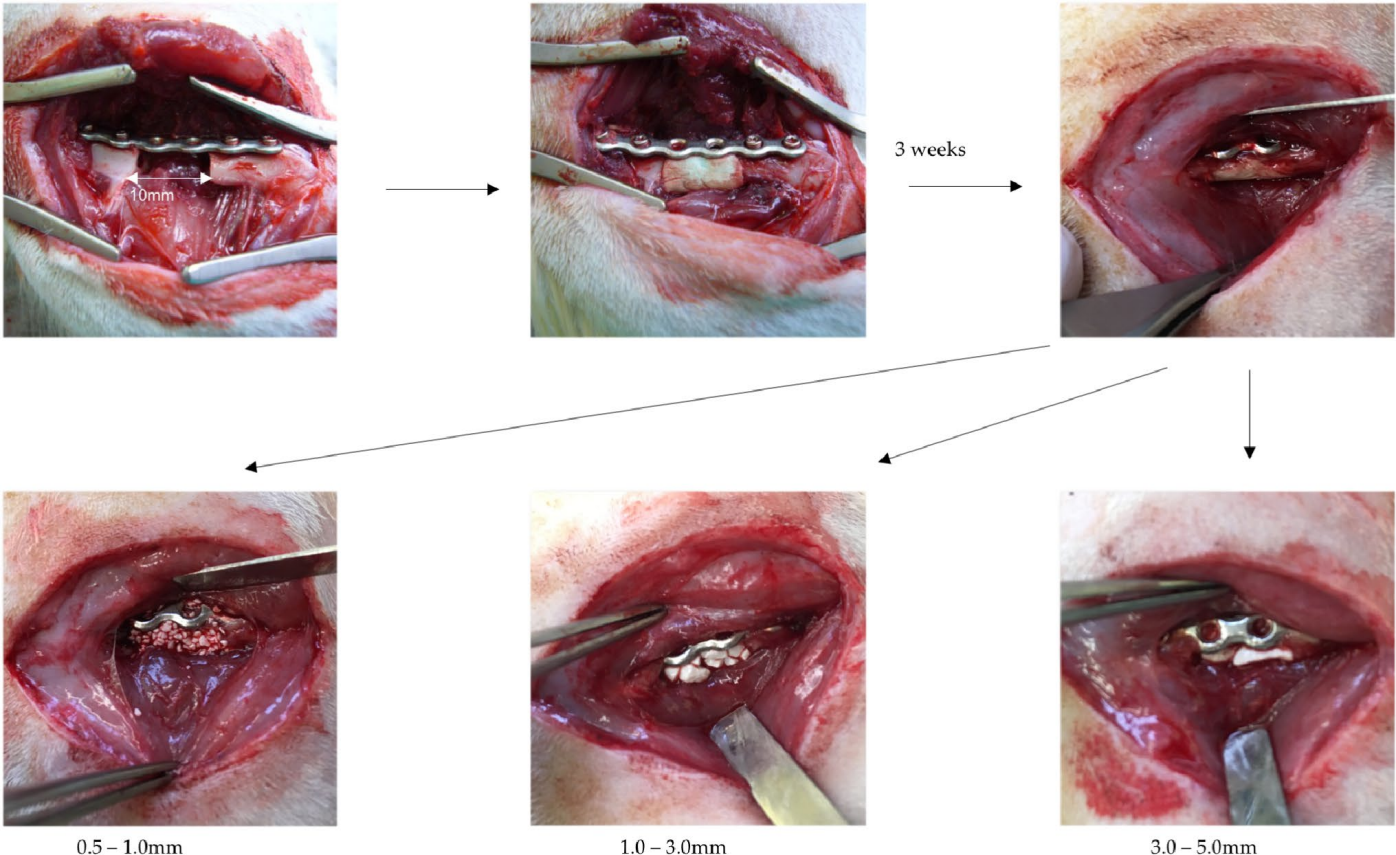
Situs with the femoral defect (10 mm), stabilized with plate osteosynthesis, filled with PMMA. Three weeks later, a membrane has developed which is opened and filled with either small, medium, or large sized Herafill^®^-granules

Three weeks after the initial operation, the induced membrane surrounding the defect/PMMA (Palacos) cement spacer was opened and the spacer was taken out and weighed. The membrane was filled with Herafill^®^-granules (small 0.5–1.0 mm/medium 1.0–3.0 mm/large 3.0–5.0 mm) or autologous cancellous bone. Afterwards, the membrane was closed with interrupted 5–0 Vicryl sutures and the wound was closed as previously described. The operative approach and the postoperative aftercare were performed as in the initial operation (Fig. 1).

Eight weeks after the second operation, animals were sacrificed with an overdose (500 mg/kg) of pentobarbital intraperitoneally administered. The femora were removed carefully, wrapped into wet gaze and stored at − 80 °C until further processing.

For histological analysis, femurs were decalcified and fixed in 10% formalin solution for later paraffin embedding.

### Treatment groups

For this study, we analyzed animals of the groups 1–4 of the study on which this project is based [14]. Animals were allocated to four groups depending on defect filling (Herafill^®^ small, medium, large and syngenous spongiosa) (Table 1). Of these groups, eight animals per group (in total *n* = 32 animals) were used for histological analysis, four additional animals were sacrificed to serve as donors for syngenic bone.

**Table 1:** Group setup

Group	Scaffold	No. of animals
1	Herafill^®^ small	8
2	Herafill^®^ medium	8
3	Herafill^®^ large	8
4	Syngenic bone	8
		= 32

### Histological analysis

For histological analysis, femurs were defrosted and fixed in 10% Zinc-Formal-Fixx (Thermo Electron, Pittsburgh) for 20 h. Subsequently, decalcification in 0.25 M Trizma base (Sigma–Aldrich) and 10% EDTA, pH 7.4, followed. Decalcified femurs were paraffin embedded and cut in Sections (3 μm) parallel to their long axis. Movat's pentachrome staining was performed as described by Garvey et al. using a staining kit (Morphisto, Frankfurt, Germany) [25].

For evaluation, the periosteum regrowth from both sides into the defect was measured and given as the sum of distances from the right and left side. A value of 7000 µm, for example, means a regrowth of 3.5 mm from right and left fracture site into the defect, leaving a 3 mm gap.

Histomorphometric analysis was used to determine the extent of bone formation in the defect area. Using the polygon tool, the bony tissue within the defect boundaries was marked and the percentage of new bone to total defect area was calculated. The software ImageJ (https://imagej.nih.gov/ij/) was used. The location of new bone formation was evaluated purely descriptive.

### Radiological analysis

Exemplarily, representative bones of each group were evaluated for new bone formation by µCT as described previously [14, 26, 27]. Two-dimensional and three-dimensional reconstructed bone defects were evaluated qualitatively. For 3D reconstruction of the bone defect, the Invesalius 3.1 (https://invesalius.github.io) software was used.

### Statistics

Results are presented as boxplots of the median, 25 and 75% quartiles, respectively. Whisker indicates minimum and maximum. Statistical analysis was done with Bias 11.02 (Epsilon-Verlag, Darmstadt, Germany). The power analysis was planned to detect differences in histological evaluation of bone mass increase (based on Movat's pentachrome staining) between four experimental groups (syngenic bone, small, medium, large granules). Based on previous comparable studies [21, 28, 29], a pooled standard deviation of 25% and a power of 80% were considered to detect true differences in means of 45% between the treatment groups at a level of two-sided significance of 5%. These considerations resulted in a group size of *n* = 8 for histology groups. Nonparametric Kruskal–Wallis test with Bonferroni–Holm corrected Conover–Iman post hoc analysis was used to compare differences between the groups. The categorial data were statistically analyzed using Fisher's exact test. *P* values below 0.05 were considered statistically significant, and *P* values between 0.05 and 0.1 were valued as a statistical trend.

## Results

### Increased periosteum regrowth with small granule size Herafill^®^ scaffold

**Fig. 2:**
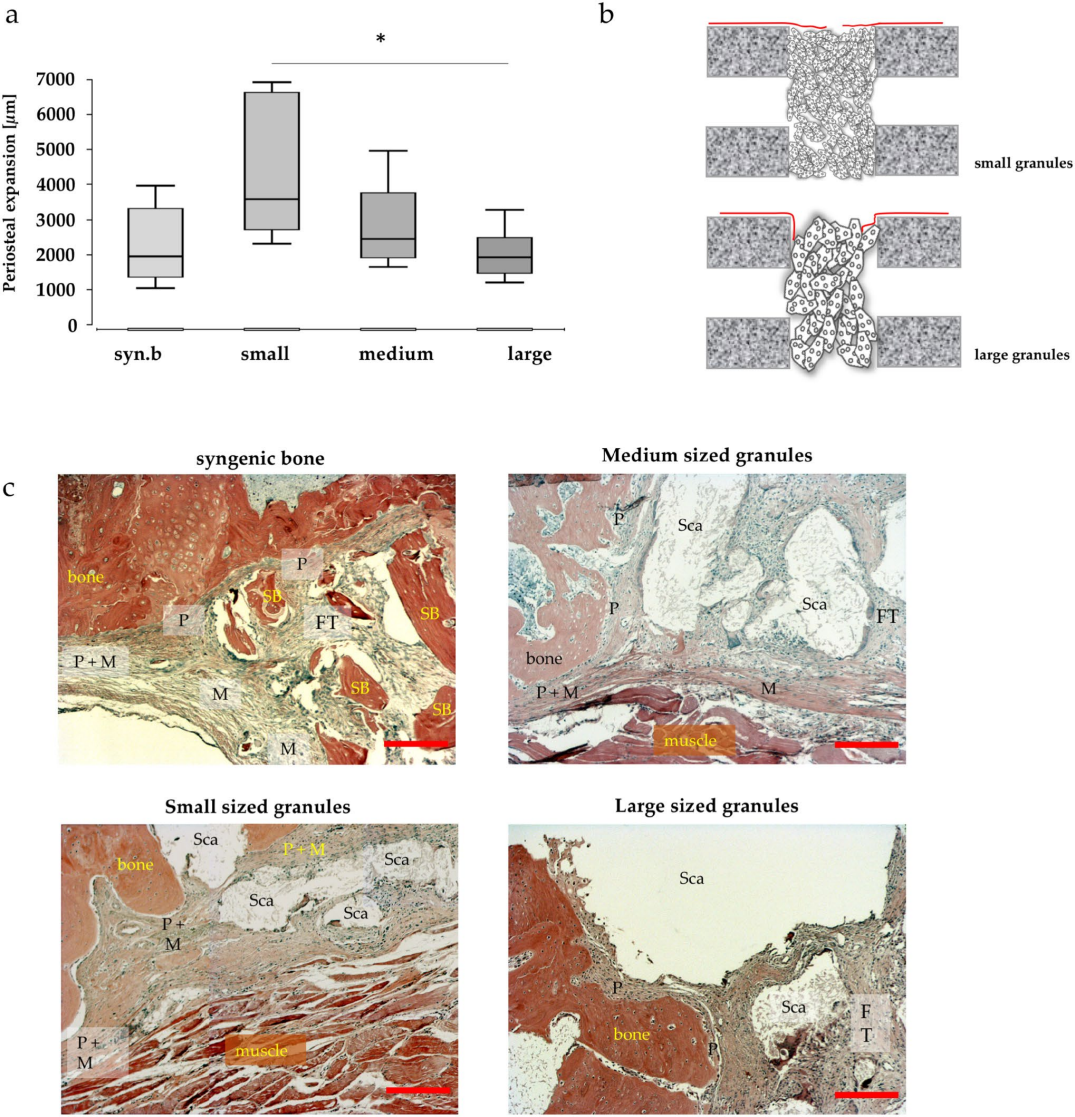
Periosteal regrowth into the defect. Significantly increased regrowth when small granules were used. Significantly less periosteal regrowth in group with syngenic bone compared to small granules (*p* < 0.05); values are given as sum of distances from the right and left side of the defect in µm and presented as boxplot **(a)**. Model of the defect filled with small respectively large granules, demonstrating deviation of periosteum into the defect **(b)**. Course of the periosteum and of the induced membrane in the bone defect depending on the size of the bone replacement granules, detailed histology (HE-staining) **(c)**. Periosteal structures originating from the periosteum are often deflected by 90° towards the bone ends when using the large granules, which probably favors the formation of bony caps and thus the development of pseudarthrosis. When the small granules are used, the periosteum-derived structure is often deflected in the extension of the cortical bone, resulting in increased bone formation in these areas. Bar: 200 µm; *FT* fibrous tissue; *M* derived from induced membrane; *P* derived from periosteum; *Sca* scaffold; *Syn. B.* syngenic bone; *small* small granules; *medium* medium granules; *large* large granules

In Movat’s pentachrome-stained histological slides, the development of periosteum regrowth from the fracture site over the defect was analyzed. An increased regrowth was found when small granules were used, reaching the level of significance when compared to large granules (*p* < 0.05). Also, defects filled with syngenic bone showed significantly less periosteal regrowth around the defect compared to defects filled with cell-free small granules (Fig. 2a).

The behavior of periosteum at the fracture site was also analyzed and it was found that occurrence of ‘bone capping’—the ingrowth of periosteal structures into the defect after deviating over the fracture site—was significantly decreased when smaller granules were used. In samples with larger granules, the periosteum tended to kink over the cortical rim, as illustrated in a representative model in Fig. 2b and histological slides of all treatment groups in Fig. 2c.

**Fig. 3:**
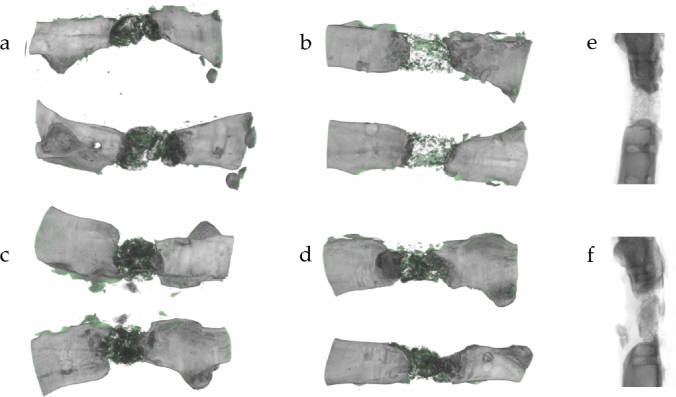
Bone formation in three-dimensional µCT of groups 1–4 **(a–d)**. Defects treated with small granules show newly formed osseous tissue (green colored) mainly in close vicinity to the induced membrane in a tubular shape **(b)**. Bone formation in defects with large granules is mainly concentrated within the defect **(d)**. Two-dimensional X-ray of bones filled with small **(e)** and large granules **(f)** shows decreased contact with partially radiused blunt fracture rim and limited connection to large granules. Small granules, in contrast, create a broad contact area

This observation was confirmed in conventional two-dimensional X-ray and three-dimensional reconstruction. Here, the interface between defect filling and fracture site showed decreased contact area with partially radiused blunt fracture rims with limited connection to large granules. In contrast, using small granules, the contact area between granules and fracture rim was broad and expanded over the complete fracture site, as shown in representative images in Fig. 3e, f.

### Increased ossification of the induced membrane after implantation of the smallest sized granules

**Fig. 4:**
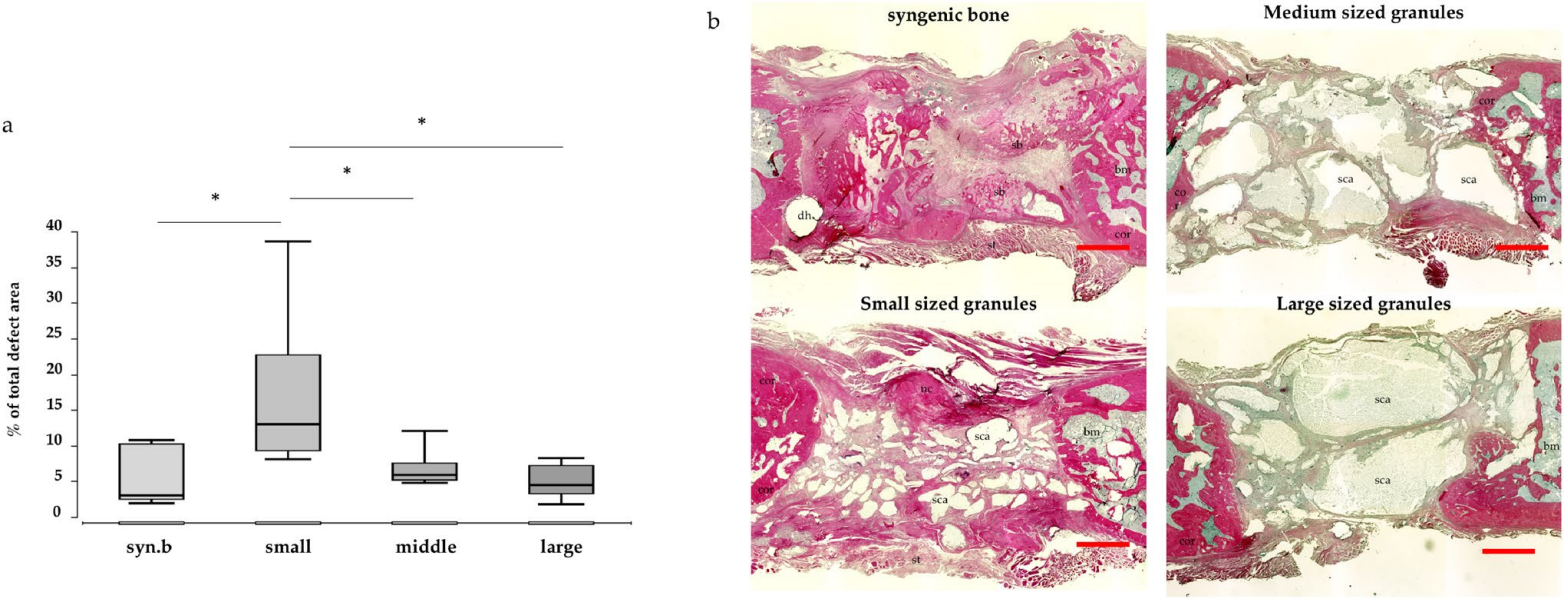
Osseous tissue in direct neighborhood to the induced membrane. Values are given as percentage of total defect area and presented as boxplot. **a** Significantly increased callus tissue when small granules are used compared to large granules and syngenic bone. Exemplary HE-stained slides of defects treated with syngenic bone, small, medium and large granules and **b** Pink stained areas indicate newly built callus, which is found increased around the induced membrane in groups with small granules. *Syn. B* syngenic bone; *small* small granules; *medium* medium granules; *large* large granules. **a** syngenic bone; **b** small granules; **c** medium granules; **d** large granules. *dh* drill hole; *sb* syngenic bone; *st* soft tissue; *bm* bone marrow; *cor* corticalis; *sca* scaffold; *nc* new callus

Callus formation was evaluated histologically in hematoxylin–eosin stained slides. New bone formation in direct neighborhood to the induced membrane was distinctly increased in bones treated with small granules (Fig. 4a). Representative images are shown in Fig. 4b.

Histological results could be reproduced in radiological evaluation. Three-dimensional µCT pictures of defects treated with small granules showed newly and tubularly formed osseous tissue, mainly allocated in vicinity to the induced membrane. In groups treated with larger granules, newly formed osseous tissue was more concentrated within the defect (Fig. 3a–d).

## Discussion

In an earlier study, we investigated the influence of the granule size of the bone graft substitute Herafill^®^ on bone healing of a critical size defect in combination with Masquelet technique [14]. Granules of the size range of 0.5–1.0 mm showed significantly improved bone healing compared to larger granule sizes, which, however, did not lead to an increased biomechanical stability in the defect zone. An impact on the vascularization in the defect zone depending on the granule size did not become evident. Small granules led to an increased accumulation of macrophages in situ, which could be assigned to the inflammatory subtype M1 by majority. Herafill^®^ granules induced an increased release of chemotactic respectively proangiogenic active factors in vitro compared to syngenic bone and β-TCP.

In a second study, further histological and radiological evaluation based on this already published study was performed. We aimed to investigate whether the particle size of a bone graft substitute using the induced membrane technique [2] has impact on the development of periosteum regrowth around the defect. Therefore, we histologically analyzed the regrowth of periosteal structures into a 10-mm femoral defect using the three different sizes of the bone graft substitute (Herafill^®^) and found significantly increased development of periosteal structures when smaller granules were used.

Although the induced membrane has oftentimes been compared to the periosteum, to the best of our knowledge, there is no data, how the periosteum itself behaves in bone defects treated with Masquelet technique.

The periosteum plays a crucial role for bone development, homeostasis, and repair [6, 30, 31]. In contrast to the induced membrane, it has a clearly definable two-layer structure; the outer layer (fibrous layer) is extensively vascularized, while the inner layer (cambium layer) provides progenitor cells (e.g. mesenchymal stem cells, preosteoblasts and osteogenic cells) for bone regeneration [7]. The periosteum provides 70–80% of the blood supply of the bone–cortex [32].

Because of its substantial qualities for bone regeneration, multiple approaches using engineered periosteum or periosteal flaps for the treatment of large bone defects are described. In an earlier study, we were able to show significantly improved bone healing treating a critical size defect with a vascularized periosteal flap [10].

Furthermore, multiple attempts at designing an artificial periosteum, tissue-engineered periosteum or a substitute have been undertaken [8]. Zhang et al. designed a biomimetic two-layered periosteum, enriched with bone marrow-derived mesenchymal stem cells and found promising results in combination with β-TCP as bone graft substitute in a calvarial bone defect of rat [33].

While Masquelet described the histological properties of the induced membrane similar to those of synovial tissue [2], Cuthbert et al. characterize the induced membrane as histologically comparable to original periosteum, but much thicker, both sharing strong architectural similarities [34].

The induced membrane is a rich source of MSCs; molecular analysis of expanded cells from induced membrane and periosteum revealed a similar RNA-profile to BM-MSC with respect to osteoprogenitor cells. SDF-1 transcript was greater in expanded cells from the induced membrane and was outlined as a difference. SDF-1 is involved in regulating MSC-trafficking; therefore, MSCs of the induced membrane are assumed to have an enhanced ability to recruit cells into the defect [34]. Due to the great parallels, Cuthbert et al. even suggest the term ‘induced periosteum’ instead of induced membrane [34].

Henrich et al. compared the induced membrane to mature periosteum in a femoral defect in rat and found significant differences [4].

Vascularization in periosteum is predominantly concentrated in the outer fibrous layer, while the cambium layer is poorly vascularized. In induced membranes, vessels are located mostly in the middle parts and the area furthest away from the PMMA. VEGF-positive cells were found in membranes, predominantly after two weeks, while the periosteum showed rarely VEGF-positive cells. STRO-1- and RUNX-2-positive MSC were found significantly more frequent in the periosteum than in induced membranes, predominantly in the cambium layer. In induced membranes, cells were concentrated along the outer surface, not in the inner regions. In contrast to the findings of Cuthbert et al., SDF-1 was not detectable, neither using immunohistochemistry nor western blotting technique [4].

While thickness and cellular content of the induced membrane change with its age [4], structure and cell population of the periosteum are also reported to be age-related and site-specific, too [5, 35]. Fan et al. compared juvenile rats (seven weeks) to aged rats (2 years) and found significantly more STRO-1, F4/80-positive cells and blood vessels in diaphyseal areas of young animals. In metaphyseal areas, thickness and cell numbers were much higher [5].

Gaio et al. compared mechanical characteristics of the induced membrane and periosteum in terms of tensile strengths and shrinking properties and found distinct differences [36, 37].

Despite their histologically and mechanically significant differences, there is a wide accordance that both—periosteum and induced membrane—promote bone healing and, in the case of periosteum’s absence or lesion, the risk for nonunion of the fracture is increased [38]. However, it is also well known that interposition of periosteum or other soft tissue into a fracture gap may lead to malunion [39, 40]. Therefore, so called ‘bone capping’—the ingrowth of periosteal structures into the defect after deviating over the fracture site—should be avoided. Our data show that the granule size of the bone graft substitute has significant influence on the development of the periosteum. Using smaller granules, the periosteum’s regrowth around the defect was most extensive (Fig. 2). An explanation for this observation is that a tighter packing and increased contact to the corticalis at the rim of the defect was achieved. This way, a lead structure exists, where periosteal structures can grow along, and bone capping was found significantly less frequent in animals treated with small granules compared to all other groups. Also, the probability for the periosteum to merge with the induced membrane is increased, which might be a benefit for bone regeneration. Using larger granules or syngenic bone, having a particle size corresponding to large Herafill^®^-granules (3-5 mm), significantly decreased regrowth of periosteum into the defect was observed.

While it has already been shown, that usage of smaller granules leads to increased callus formation within the defect and between the single granules [14], we could now prove that also formation of osseous tissue in direct neighborhood to the induced membrane is distinctly increased in animals treated with the smallest granules (Fig. 4). This histological observation could be confirmed by µCT images as exemplarily shown in Fig. 3. This observation could be affiliated to the significantly increased regrowth of periosteal structures, which supports bone formation by its rich vascularization and as a source of stem cells. This way, bone healing from inside the defect zone is supported by an increased mineralization of periosteum from outside the defect.

As a clinical impact of this observation on the treatment of large bone defects with the induced membrane technique, it might be considered whether smaller granules of a bone graft substitute could be used in the peripheral areas of a defect to optimize the interface with the fracture site and avoid bone capping.

As a limitation of this study, the question must be considered as to whether the composition of Herafill^®^ might be a reason for the observations described above and if other scaffolds such as β-TCP (Chronos) might have brought out similar results. Another interesting aspect which has not been examined here would be how the periosteum develops in older animals, as there are significant age-related differences reported concerning structure and cellular content of the periosteum. Also, we did not achieve a mechanically stable defect bridging after a healing period of 8 weeks. One aspect for further investigation could be, if a longer observation time would bring out increased mineralization. Those might be aspects for further investigation. However, to the best of our knowledge, there are no data published about the periosteum’s behavior in defects treated with the induced membrane technique.

In conclusion, it can be stated that the granule size of the bone graft substitute Herafill^®^ in combination with Masquelet technique has significant impact on periosteum regrowth around the defect zone. Granules of the size range of 0.5–1.0 mm showed significantly increased periosteum regrowth into the defect zone compared to larger granules or syngenic bone, which might be ascribed to an improved interface between granules and the fracture site achieved by a tighter packing. Increased regrowth of periosteal structures leads to significantly increased formation of osseous tissue in direct neighborhood to the induced membrane, thus supporting bone healing of the defect.
